# Effectual visible light photocatalytic reduction of para-nitro phenol using reduced graphene oxide and ZnO composite

**DOI:** 10.1038/s41598-023-36574-7

**Published:** 2023-06-12

**Authors:** Sasireka Velusamy, Anurag Roy, Ezrah Mariam, Satheesh Krishnamurthy, Senthilarasu Sundaram, Tapas K. Mallick

**Affiliations:** 1 Solar Energy Research Group, Environment and Sustainability Institute (ESI), Faculty of Environment, Science and Economy, Penryn Campus, University of Exeter, Cornwall, TR10 9FE UK; 2 School of Engineering and Innovation, The Open University, Milton Keynes, MK7 6AA UK; 3 Cybersecurity and Systems Engineering, School of Computing, Engineering and the Built Environment, Edinburgh Napier University, Edinburgh, EH10 5DT UK

**Keywords:** Nanoscale materials, Environmental sciences, Environmental chemistry, Environmental impact

## Abstract

Removing wastewater pollutants using semiconducting-based heterogeneous photocatalysis is an advantageous technique because it provides strong redox power charge carriers under sunlight irradiation. In this study, we synthesized a composite of reduced graphene oxide (rGO) and zinc oxide nanorods (ZnO) called rGO@ZnO. We established the formation of type II heterojunction composites by employing various physicochemical characterization techniques. To evaluate the photocatalytic performance of the synthesized rGO@ZnO composite, we tested it for reducing a common wastewater pollutant, para-nitro phenol (PNP), to para-amino phenol (PAP) under both ultraviolet (UV) and visible light irradiances. The rGO_x_@ZnO (x = 0.5–7 wt%) samples, comprising various weights of rGO, were investigated as potential photocatalysts for the reduction of PNP to PAP under visible light irradiation. Among the samples, rGO_5_@ZnO exhibited remarkable photocatalytic activity, achieving a PNP reduction efficiency of approximately 98% within a short duration of four minutes. These results demonstrate an effective strategy and provide fundamental insights into removing high-value-added organic water pollutants.

## Introduction

Modern industrialization has led to an increase in the utilization of phenolic compounds such as nitrophenolic and other aromatic compounds. These compounds have found wide-ranging applications in various industries, including textiles, rubber, paints, fertilizers, explosives, curing agents, food industries, and antioxidants^1^. Pollutants from these industries are the creator of the carcinogenic effect on the ecological system^2,3^. Eventually, harmful industrial discharges and agricultural pesticide applications seep through the soil, potentially contaminating the soil and environmental groundwater sources^4,5^. Normally, phenols form various derivatives in the water; among them, para-nitro phenol is a toxic industrial pollutant that discharges from dyes, pesticides, plasticizers, pharmaceuticals and agrochemicals industrial waste^6–8^. Roughly, 27% of para-nitrophenol (PNP) is utilised in manufacturing pesticides (parathion) and 13% is used in synthesising dye components. The maximum accepted concentration of nitro phenols in portable water is 0.5 μ mol L^−1^. PNP has been identified as a priority pollutant by the United States Environmental Protection Agency (USEPA) due to its carcinogenic properties. As a result, the concentration of PNP in natural water bodies should be kept below a certain threshold (< 10 mg L^−1^)^9,10^. PNP is a potent neurotoxin that can cause severe damage to the central nervous system, liver, and kidneys, even at low concentrations in humans, as reported by various studies^11–13^. Inhaling or ingesting PNP in the short term can result in a range of health issues, including headaches, drowsiness, nausea, cyanosis, and eye irritation. Moreover, due to its high stability, low biodegradability, and water solubility, PNP has the potential to cause harm to ecosystems^2^.

Visible light photocatalysts offer a promising solution for environmental remediation and sustainable wastewater treatment. One of the main advantages of using a visible light photocatalyst is that it utilizes a broader range of the electromagnetic spectrum, which is more energy-efficient and cost-effective than using ultraviolet (UV) light. Visible light is also less harmful to the environment and human health, making it a safer option for photocatalytic applications. Furthermore, visible light photocatalysts are more effective for degrading organic pollutants such as dyes, pharmaceuticals, and pesticides, which are prevalent in industrial wastewater.

A composite photocatalysts offer advantages over single-component photocatalysts, such as improved photocatalytic efficiency, enhanced light absorption, and reduced recombination of electron–hole pairs. The combination of materials can also broaden the spectral response range of the photocatalyst, allowing for more efficient utilization of light energy. Furthermore, the use of composite photocatalysts can improve the stability and durability of the photocatalytic system.

In this scenario, a semiconductor with heterojunction rGO@ZnO composite photocatalyst was developed to mineralise the organic pollutant PNP at the disbursal of ultra-violet (UV) and visible light irradiations^14–16^. Among the various ZnO nanostructures, nanorods have attracted considerable attention because of their high stability and large specific surface area^17^. The benefits of utilising the one dimensional (1D) nanostructures like nanorods can provide a higher surface area to volume ratio and enable efficient carrier transport in comparison with zero and other dimensional nanostructures due to the decreased boundaries, surface defects and structural disorders^18^. However, utilisation of ZnO in photocatalysis exhibits some disadvantages namely, (i) particle aggregation during photocatalysis activity that restrains the activity of ZnO on a large scale, (ii) the restriction of ZnO usage in the visible region because of its wide band gap, (iii) the expeditious recombination of charge recombination of the photo generated electron–hole pairs^19,20^. Further, the wide band gap restricts the catalytic activity due to the low light absorption, to conquer this issue the band gap of ZnO can be tuned by ion doping or coupling with different semiconductors, deposition metals and non-metals, defect Engineering and co-catalyst will regulate the band gap energy^21,22^. Multiple studies have been reported on ZnO combinational catalysts such as NiS/ZnO^23^, CuO/ZnO^24^, Ni/rGO^25^, Ag/ZnO^26–28^, ZnO^29^, graphene-ZnO^30,31^, Co–ZnO^32^ for the PNP reduction reaction. Recently, Bekru et al., reported the (rGO–ZnO)/CuO nanocomposite interface is a highly efficient photo-catalyst for PNP reduction^33^. Reduced graphene oxide and semiconductor based composite exhibit increased photocatalytic performance than pure oxide nanostructure^34^. Functional groups like hydroxyl (–OH), carboxyl (–COOH), epoxide (C–O–C) and carbonyl (C=O) on the GO surface, make a strongly hydrophilic nature, along with greater stability while dispersing with water^35^. Its low energy band gap (1.17 eV) makes its excellent optical properties for a diverse range of applications^36^. The rGO@ZnO hybrid composite is expected to reduce the recombination of charge carriers and increase the photocatalytic efficiency where rGO acts as an electron acceptor and obstructs the electron–hole recombination^37^. This study showcases the development of a composite photocatalyst, rGO@ZnO, for the mineralization of the organic pollutant PNP under both UV and visible light irradiation. The work presents a straightforward strategy for developing visible light photocatalysts that can be scaled up to industrial processes, making them cost-competitive with existing technologies. The degradation process was optimized and analyzed by determining degradation kinetics, and catalyst recyclability was examined for the stability of the photocatalyst.

## Results and discussion

### Phase analysis

The rGO_x_@ZnO (x = 0.5–7 wt%) composites were synthesized by varying the weight of rGO and ZnO. The XRD analysis revealed the involvement of rGO sheets on ZnO during composite formation and showed that the crystallinity of the ZnO rods were significantly enhanced by the addition of a higher amount of rGO. The maximum enhancement was observed for the rGO_5_@ZnO composite. The crystallinity enhancement of ZnO rods can be attributed to their adequate folding with rGO on ZnO, which leads to discreet rGO_x_@ZnO composites. The XRD patterns of the 3,5 and 7.0 wt% of rGO_x_@ZnO samples were shown in Fig. S1.

**Figure 1:**
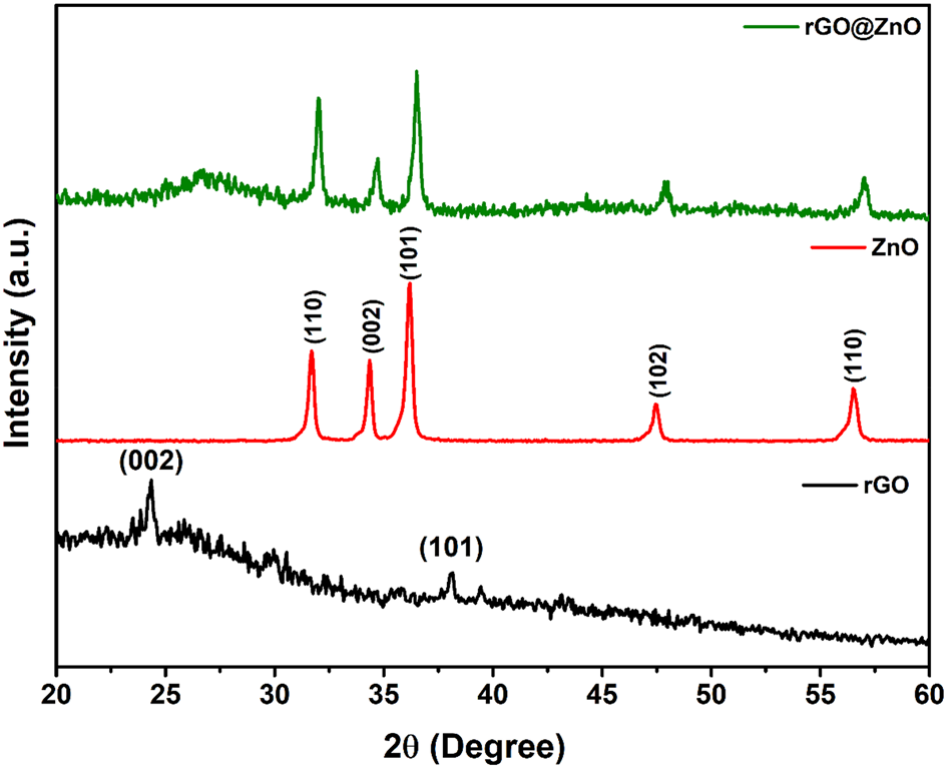
XRD patterns of rGO, ZnO and rGO_5_@ZnO composite samples.

The powdered XRD results for the synthesized rGO_5_@ZnO composite were compared with synthesized ZnO and rGO, as shown in Fig. 1. The XRD pattern of ZnO shows the diffraction peaks positioned at 2θ values of 31.5°, 34.4°, 36.3°, 47.3°, and 56.4° could be indexed to (100), (002), (101), (102) and (110) lattice planes of hexagonal wurtzite structure, respectively. All the peaks mentioned above are agreed well with the standard data sheet with the standard JCPDS No. 36-1451^38^. The diffraction peaks of ZnO and rGO@ZnO were similar in appearance, indicating the formation of a composite structure. The absence peak of rGO (002) at 24.8° indicated that the surface of the rGO was covered with ZnO comprehensively, whereas rGO cannot be observed over the indexed peaks of wurtzite ZnO. The rGO@ZnO heterojunction retained all the diffraction peaks of ZnO with weakened intensity as the amount of rGO increased. The XRD analysis showed that the average crystallite size of ZnO was ~ 32 nm.

### Optical analysis

Further structural information on the rGO@ZnO composite was obtained using Raman Spectroscopy and compared with rGO and ZnO. As shown in Fig. S2a, characteristic peaks at 332, 380, 436, and 583 cm^-1^ were observed, corresponding to A_1_, A_1_ (LO), E_2_ (high), and E_1_(LO) vibrational modes of ZnO hexagonal structure, respectively. The E_2_ high mode in ZnO is associated with the vibration of oxygen atoms in the ZnO lattice and is characteristic of the peak of the hexagonal wurtzite structure^39^. On the other hand, rGO in Fig. S2b displayed peaks at 1351, 1564, and 2896 cm^-1^, attributed to the first-order D-band (internal structure defective or disordered sp^3^ carbon), G-band (ordered band of graphite), and 2D-band (second order of D-band but no defects), respectively. The G-band is assigned to all sp^2^-bonded carbons, providing further information on the in-plane vibration of sp^2^ bonded carbon^40^. The intensity of the D band and G band reflects the stacking and the number of layers in rGO nano sheets, with an intensity ratio of D-band to G-band (I_D_/I_G_) of 0.86 in multilayer rGO.

**Figure 2:**
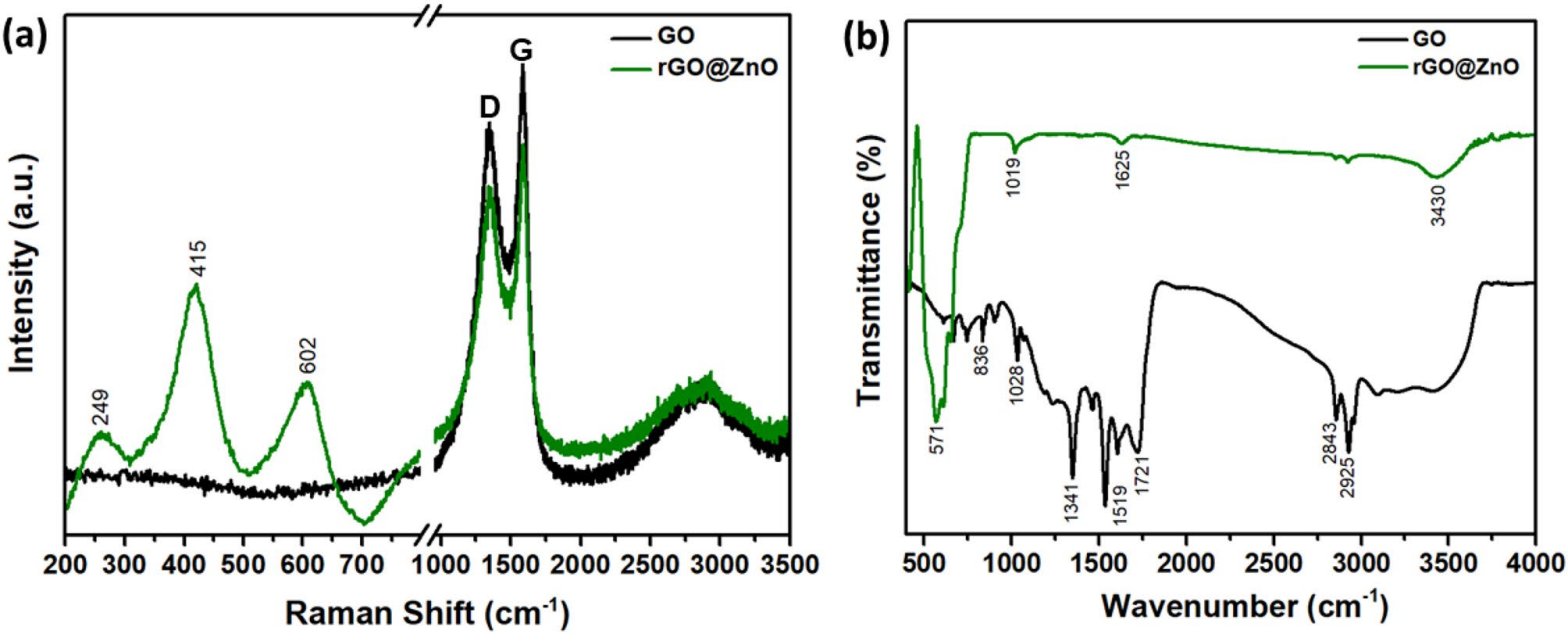
(**a**) Raman and (**b**) FTIR spectra of rGO_5_@ZnO composite, which is compared with bare GO.

In Fig. 2a, the combined characteristic Raman peaks of both ZnO and rGO in the composite confirm the successful formation of rGO_5_@ZnO. Moreover, the rGO_5_@ZnO composite exhibits a significant reduction in the characteristic G-band compared to D-band when compared to GO. The spectrum of rGO_5_@ZnO composite indicates that the stretching band vibration of C-H molecules overlapped in the D band of the rGO layer during composite formation, making the D band broad. Additionally, the reduction of the G-band during the composite formation suggests that in-plane graphitic sp^2^ bonds are attached to ZnO, consistent with previous reports. The ratio of the Raman intensity between the D and G bands (I_D_/I_G_) can be used to characterize the extent of reduction in GO.

The I_D_/I_G_ ratio of the composite (0.883) was lower than that of pure rGO (0.894). This is attributed to the emergence of oxygen functional groups from GO during the composite formation process, leading to a decrease in the sp^2^ domains. The D and G bands in the Raman spectrum correspond to the conversion of sp^2^-hybridized carbon to sp^3^-hybridized carbon (indicating the presence of defects) and the sp^2^ carbon network, respectively. The shift or broadening of Raman peaks or appearance of new peaks may be associated with the presence of oxygen vaccancies in catalytic materials^41^. Furthermore, led to the collapse and conversion of the planar sp^2^ carbons into the sp^3^ carbons, the composite exhibited a reduced crystallite size compared to bare GO, as evidenced by the broadening Raman peak area. The lower I_D_/I_G_ ratio can be attributed to the presence of oxygen-containing functional groups and the interaction between the rGO sheet and ZnO nanorods.

FTIR spectroscopy was utilized to investigate the functionality and interaction present in the rGO_5_@ZnO composite, and compared with GO alone. As shown in Fig. 2b, the typical FTIR spectrum of GO exhibits characteristic bands of oxygen-containing functional groups, such as C-O alkoxy (1028 cm^-1^), C–O epoxy (1192 cm^-1^), C–O stretching (1341 cm^-1^), and C=O carbonyl (1721 cm^-1^), indicating the complete oxidation of graphite to GO during the Hummers method. The bands at 2843 and 2925 cm^-1^ correspond to the methylene group (-CH_2_) of the graphitic network of the GO. In contrast, in the spectrum of the rGO_5_@ZnO composite, the oxygen-containing vibrational bands were significantly reduced, indicating a significant reduction of GO under hydrothermal treatment. The bands at 3430 and 1625 cm^-1^, corresponding to the stretching and bending vibration of –OH, respectively, were also significantly reduced and almost disappeared once the composite formed. The broad band in the range of 500–900 cm^-1^ can be attributed to the combination of Zn–O–Zn and Zn–O–C stretching vibrations, indicating the strong chemical coupling between ZnO and rGO in the composite. These results support the successful reduction of GO to rGO and the formation of the rGO_5_@ZnO composite.

**Figure 3:**
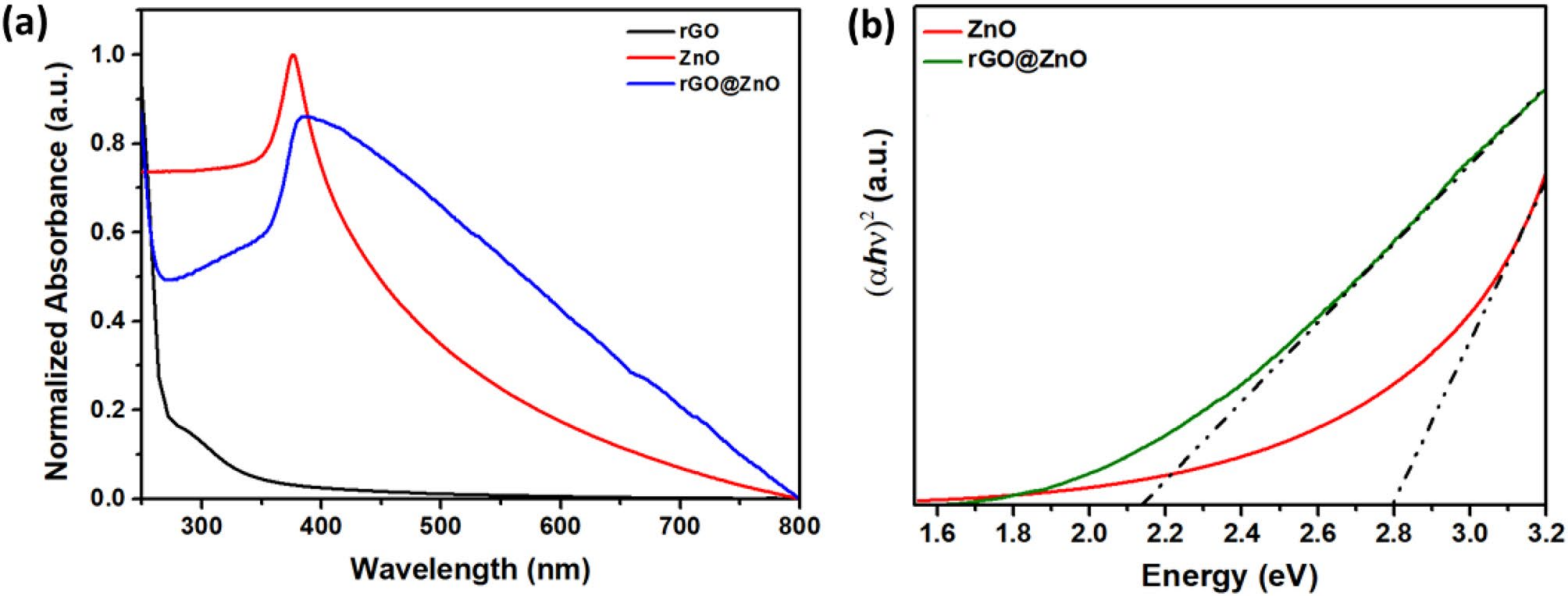
(**a**) UV–visible absorption spectra of the rGO_5_@ZnO composite compared with ZnO and rGO, (**b**) Tauc plots of rGO_5_@ZnO composite, compared with ZnO.

Figure 3a shows the results of UV–Vis absorption spectra of rGO, ZnO, and rGO_5_@ZnO composite. It was found that rGO had a weak absorption broad band at approximately 300 nm, which corresponded to the Π-Π* interaction of the graphene framework. The synthesized ZnO showed an absorption peak at approximately 370 nm, indicating its indirect band gap. On the other hand, the rGO_5_@ZnO composite exhibited broader absorption characteristics with an extended absorption edge to the visible region, resulting in the highest absorption at approximately 385 nm. This tendency of visible absorption can extend the optical response and facilitate excitation of photoelectrons, which is beneficial for its photocatalytic activity under the solar spectrum region. Furthermore, it was observed that broader absorption characteristics could enhance the indirect band gap energy of the rGO_5_@ZnO composite, which was calculated using Tauc’s Eq. (1).

1 $$\left( {\varepsilon {\text{h}}\nu } \right) = {\text{C }}\left( {{\text{h}}\nu {-}{\text{Eg}}} \right)^{{\text{n}}}$$

where C is a constant, ε is molar extinction coefficient, Eg is the average band gap of the material and n depends on the type of transition. The estimated optical bandgap of the rGO_5_@ZnO composite was found to be 2.13 eV, significantly lower than that of ZnO nanorods (2.80 eV) as shown in Fig. 3b. The band gap narrowing indicates more efficient solar spectrum utilisation and consequently more photoinduced charge generation, which contributes to the superior photocatalytic performance of the composite over native ZnO nanorods.

### TEM analysis

**Figure 4:**
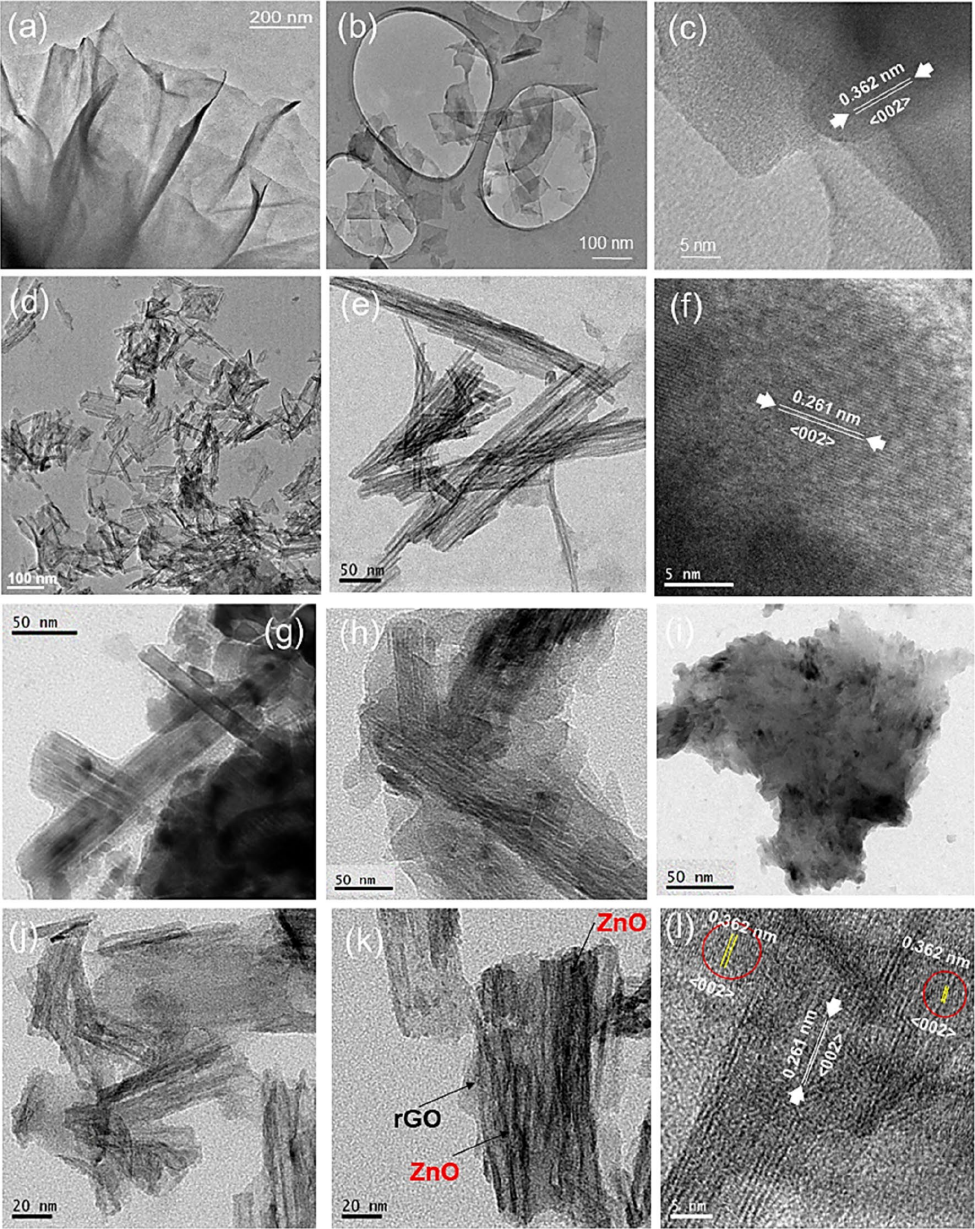
TEM bright-field images of (**a**, **b**) rGO, (**d**, **e**) ZnO, (**g**–**i**) rGO_x_@ZnO composites where x = 3, 5 and 7 wt% of rGO samples, respectively, (**j**)-(**k**) rGO_5_@ZnO composite, and corresponding HRTEM images (**c**) rGO, (**f**) ZnO, (**i**) rGO_x_@ZnO (**l**) rGO_5_@ZnO composites samples, respectively.

The morphological characteristics of pure ZnO, rGO, and rGO_5_@ZnO composite were examined using TEM, as illustrated in Fig. 4. At different magnifications, Fig. 4a and b display wafer-type structures of rGO with micrometric dimensions consisting of submicron-sized rGO sheets. Figure 4c depicts the corresponding HRTEM image of the amorphous rGO’s most intense plane, 002, with a lattice distance of 0.362 nm. Figure 4d and e exhibit the rod-like structure of synthesized ZnO samples, with nanorods' length found to be ~ 100 ± 10 nm and the average width corresponding to ~ 35 nm. In the HRTEM image of Fig. 4f, the high crystalline (002) plane of the synthesized nanorods was shown, having a lattice distance of 0.261 nm. Additionally, these rods were laterally self-assembled to form a bundle of rods. Each bundle contained similar thin nanorods tightly held together, as Fig. 4f illustrated. Figure 4g–i displayed bright-field images of the rGO_x_@ZnO composites for 3, 5, and 7 weight percentages of rGO samples, indicating that spatially interconnected rGO nanosheets occupied the exterior portion of ZnO nanorods and extended to form a wrapped morphology, revealing the formation of a heterostructure of rGO_x_@ZnO composites. The extent of rGO wrapping was effectively increased for higher rGO-contented samples. In the rGO_7_@ZnO composite, insignificant ZnO rods were observed (Fig. 4i). Consequently, the rGO_5_@ZnO sample was selected for further study, considering an optimum phase development, crystallinity, and effective morphological distribution parameters. Figure 4j and k displayed bright-field images of the rGO_5_@ZnO composite, where the ZnO nanorods were randomly distributed on rGO sheets rather than being wrapped with them. Additionally, the TEM analysis confirmed that the rGO sheets overlapped and formed a three-dimensional lattice structure on ZnO nanorods, providing faster electron mobilization under visible light. The high-resolution transmission electron microscopy (HRTEM) of the rGO_5_@ZnO composite heterostructure in Fig. 4l revealed moderate crystallinity, showing a combination of both rGO and ZnO planes. The extent of rGO wrapping to form the heterostructure composite also reduced the agglomeration of ZnO nanorods.

The introduction of rGO into (ZnO rods presents a notable opportunity to enhance light absorption in the composite material by exploiting light scattering from the disordered structure. This phenomenon extends the optical path traveled by incident light upon interaction with the nanostructures, resulting in increased absorption efficiency. Moreover, the incorporation of oxygen vacancies expands the absorption range from the ultraviolet (UV) to the visible spectrum. This effect arises from the creation of localized states below the conduction edge. The obtained findings not only provide valuable insights into the structural characteristics and properties of the composite photocatalyst but also highlight its potential for effective and sustainable wastewater treatment applications.

### XPS analysis

**Figure. 5:**
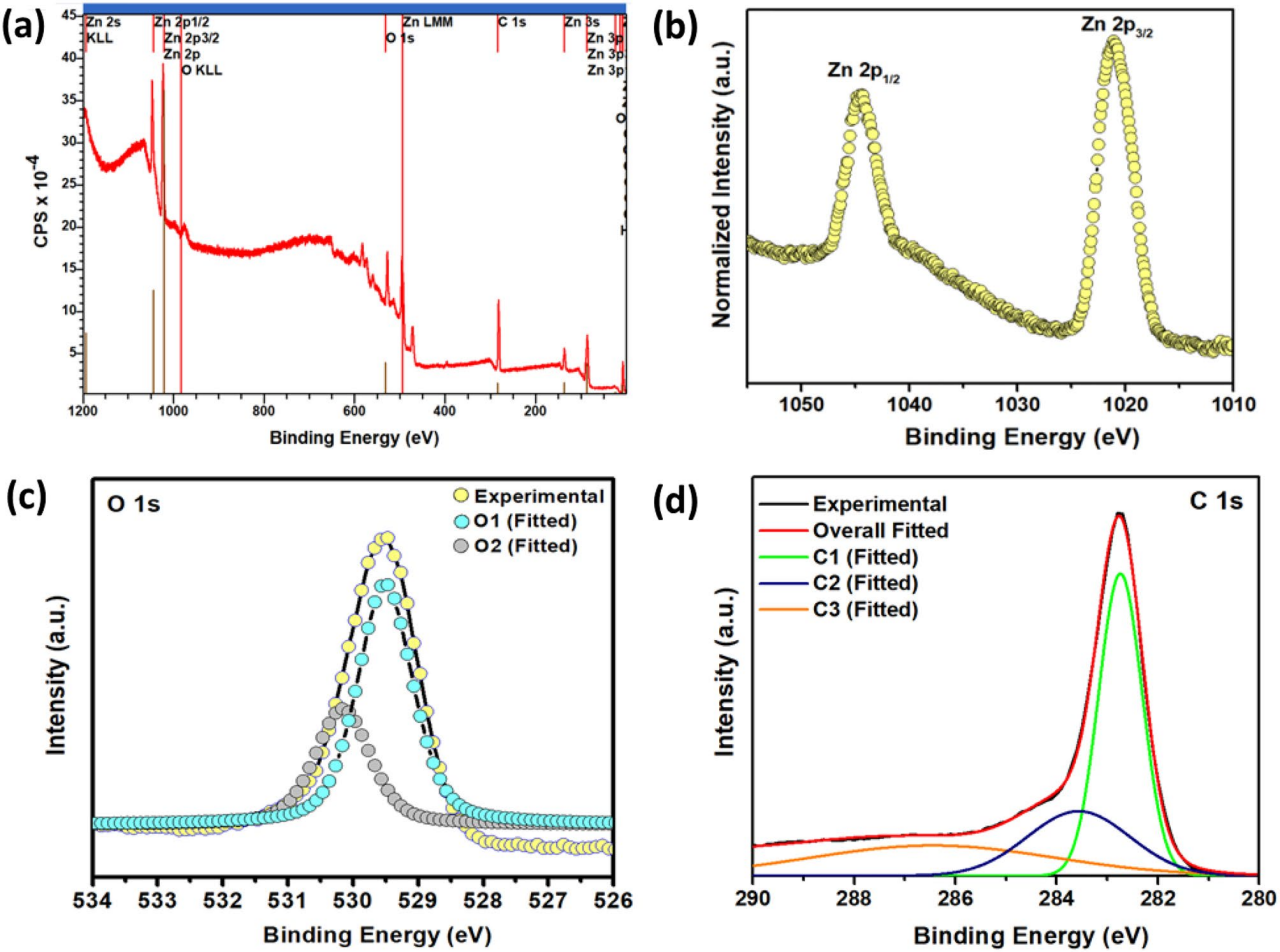
The core-level XPS spectra of the rGO_5_@ZnO composite, including the (**a**) survey spectrum and the corresponding spectrum of (**b**) Zn 2p, (**c**) O 1 s (**c**), and (**d**) C 1 s, respectively.

The survey spectrum of the rGO_5_@ZnO composite (Fig. 5a) indicates the presence of Zn, O, and C elements. Notably, all the binding energies were calibrated using contaminant carbon (C 1 s = 283.4 eV) as the reference. Interestingly, the core-level binding energies of Zn, O, and C during composite formation were blue-shifted compared to native ZnO. The Zn 2p spectrum of the composite consists of the spin–orbit split peaks of Zn 2p_1/2_ and 2p_3/2_ appearing at approximately 1044.8 and 1021.6 eV binding energies, respectively, as shown in Fig. 5b ^42^.

However, it should be noted that the energy difference between the two core levels of both samples was found to be 23.1 ± 0.2 eV, which matches well with the reported value for Zn^2+^ . A spectral deconvolution of the asymmetric O 1 s spectrum of the sample in Fig. 5c reveals three components appearing at binding energies of 530.32 and 531.74. Based on the findings of previous investigators, it can be inferred that the O1 component on the lower binding energy side of the O 1 s spectrum corresponds to lattice oxygen (O^2^^-^) ions in Zn–O bonds of the wurtzite structure (Fig. 5c)^43^. The O2 component is associated with the O^2-^ ions in oxygen-deficient regions within the ZnO matrix. Similarly, the C 1 s core-level spectrum of the composite exhibits a regular shifting phenomenon, as shown in Fig. 5d. The binding energy peak at 282.73 eV (C1) indicates a graphitic structure (C–C) corresponding to rGO. The shoulder peak at 283.61 and 286.70 eV is derived from C–OH (epoxy/hydroxy) and oxygen-containing group, O–C= , respectively^44^ . It is anticipated that the blue-shifting binding energies of the prepared composite occurred due to the chemical nature of the neighboring atoms on an individual surface during the hydrothermal treatment^45^. Also, the binding energy varies with the change in the shielding effect^46^. Additionally, the peak positions of the XPS analysis demonstrate the successful formation of the rGO@ZnO composite compared to native ZnO.

### rGO_x_@ZnO (x = 0.5–7 wt%) composite catalytic performance evaluation under the different light source

**Figure 6:**
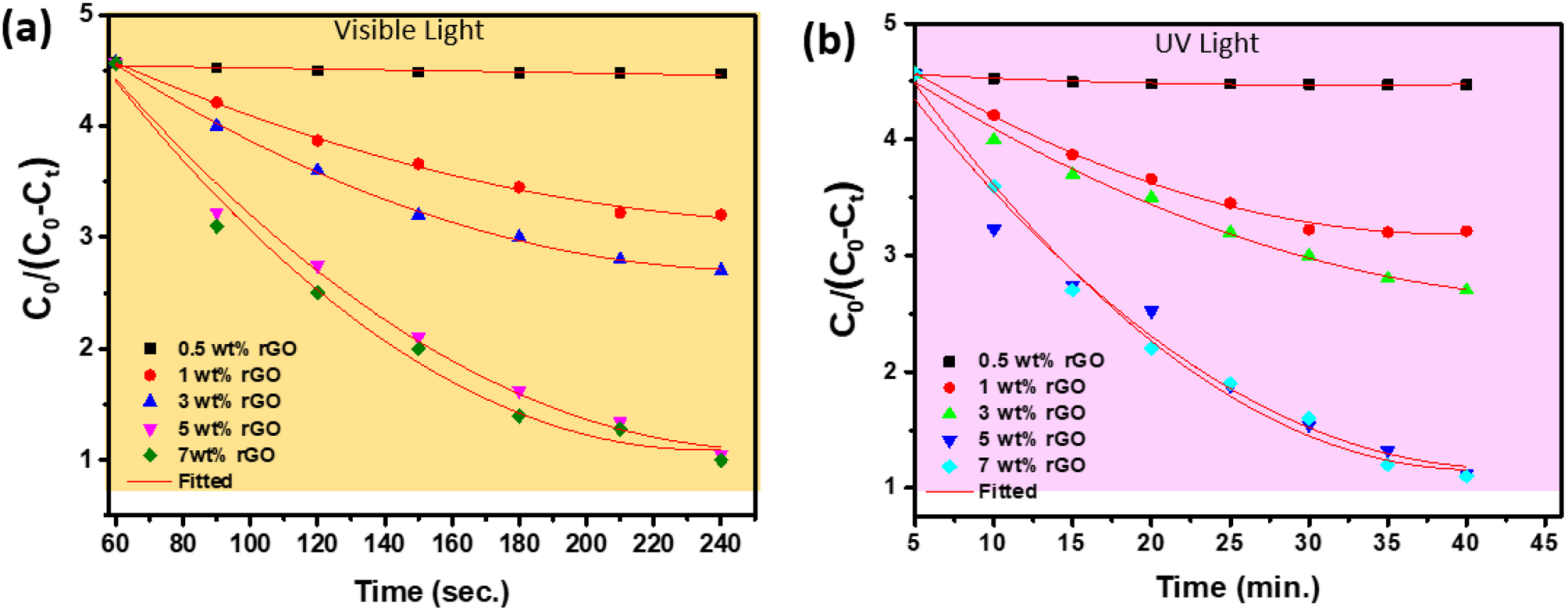
Photocatalytic reduction curve for rGO_x_@ZnO (x = 0.5–7 wt%) composite samples under (**a**) visible and (**b**) UV light for PNP, respectively.

Within the rGO@ZnO composite structure, photo-generated charge carriers can be transferred. Defects, whether naturally occurring in the structure or mechanically made, serve as charge carrier adsorption sites. Thus, the induced electrons can be transferred to the active sites and prevent the recombination of photo-generated electron (e^-^) and hole (h ^+^) pairs. To evaluate the photocatalytic degradation activity, rGO_x_@ZnO (x = 0.5–7 wt%) samples with different weights of rGO were synthesized and tested individually under UV and visible light (Fig. 6). rGO can absorb some UV light, leading to a competition in light harvesting between ZnO and rGO with increased rGO. Furthermore, excessive rGO can act as a recombination center rather than providing an electron pathway, leading to a decrease in photocatalytic performance. In contrast, ZnO generates oxygen vacancies at the grain and surface boundaries, where nanoparticle growth is impeded, leading to the creation of a stress field that acts as scattering centers for electrons and holes. This reduces the recombination rate of hole-electron pairs, thereby enhancing photocatalytic activity. Interestingly, the presence of ZnO alone did not significantly degrade PNP under UV or visible light, as illustrated in Fig. S3. This suggests that PNP degradation is not readily achieved by ZnO alone.

The rGO_x_@ZnO composite exhibits a more rapid reduction rate than its counterparts. The type of light source used for illumination has a significant impact on the reduction of 4-nitrophenol to 4-aminophenol, particularly in terms of the overall conversion duration. The rGO_5_@ZnO composite sample was found to have the highest reduction efficiency within a similar time frame compared to other compositions. When illuminated with visible light, the photocatalytic degradation occurred at a comparatively faster rate (within 4 min) with an efficiency reduction of approximately 98% (Fig. 6a). Conversely, under UV light, a similar reduction process took approximately 45 min with a reduction efficiency of approximately 60% (Fig. 6b). Thus, the heterostructured composites exhibited higher efficiencies compared to the single component.

The catalytic performance of rGO@ZnO composite (5 wt%) was evaluated in the reduction of PNP to para-amino phenol (PAP) using NaBH_4_ as the source of hydrogen. The selection of reducing agents and hydrogen sources plays a critical role in converting PNP to PAP. In this regard, hydrogen (H_2_) can be generated through NaBH_4_, as indicated by Eq. 2.

2 $${\text{BH}}_{4}^{ - } + 2{\text{H}}_{2} {\text{O}} \to {\text{BO}}_{2}^{ - } + 4{\text{H}}_{2}$$

**Figure 7:**
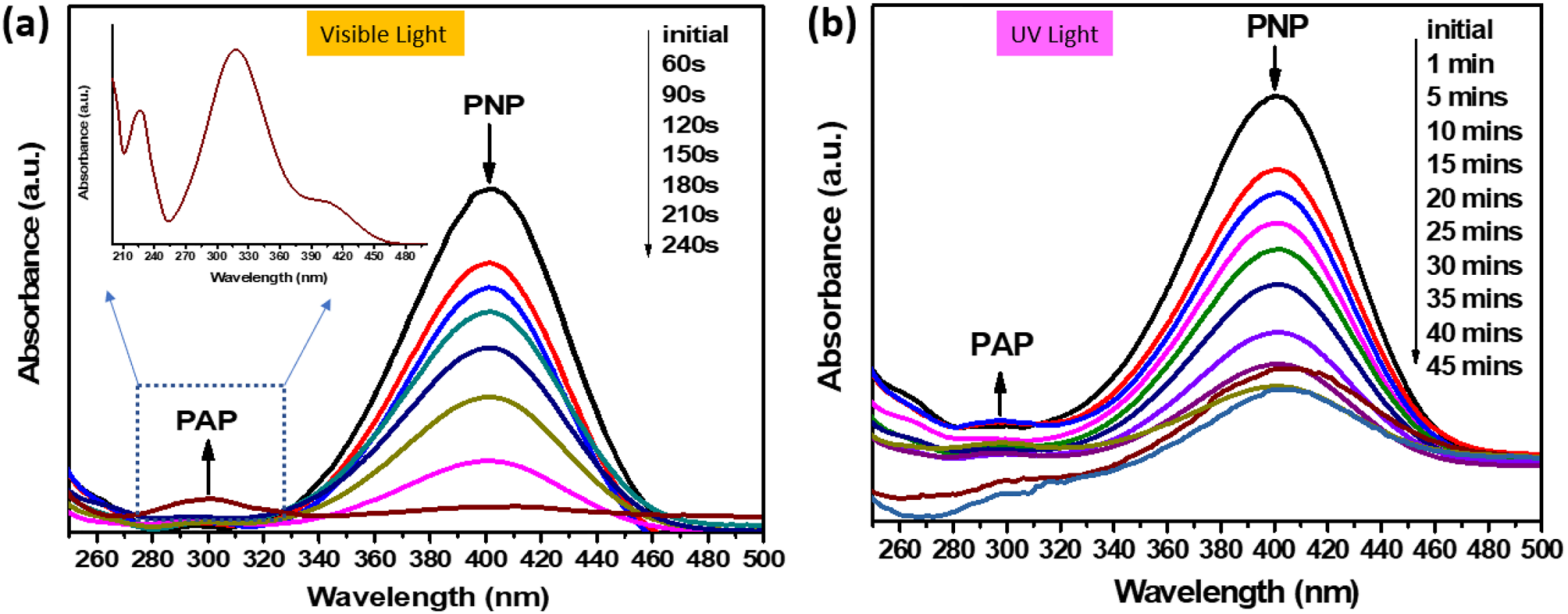
Photocatalytic reduction characteristics of PNP under (**a**) visible, and (**b**) UV light conditions, respectively.

The alkaline PNP and NaBH_4_-containing solution were mixed with the rGO_5_@ZnO composite. The addition of NaBH_4_ caused a slight yellow color change in the PNP solution, indicating the formation of PNP. The solution gradually turned dark in color, but the color faded after adding the rGO_5_@ZnO composite. To monitor the progress of the reaction, the absorption spectrum of PNP and PAP was measured at different time intervals. Initially, the UV–vis absorption spectra of the mixture showed a maximum at ~ 400 nm, attributed to the nitro compound. This was followed by a gradual decrease in absorption peaks, and a new peak at ~ 300 nm was observed, indicating the reduction of PAP. The reaction kinetics, monitored by the time-dependent absorption spectra, showed that the absorption peaks corresponding to PNP and PAP decreased consecutively, indicating the reduction of PAP. Under visible light, the intensity of the absorption peak of PNP was completely saturated in ~ 240 s (~ 4 min), indicating the success of the catalytic reduction process, as depicted in Fig. 7a. However, under indoor UV light conditions, the new peak at 300 nm, corresponding to PNP, was observed after almost 45 min, indicating incomplete degradation, as shown in Fig. 7b.

**Figure 8:**
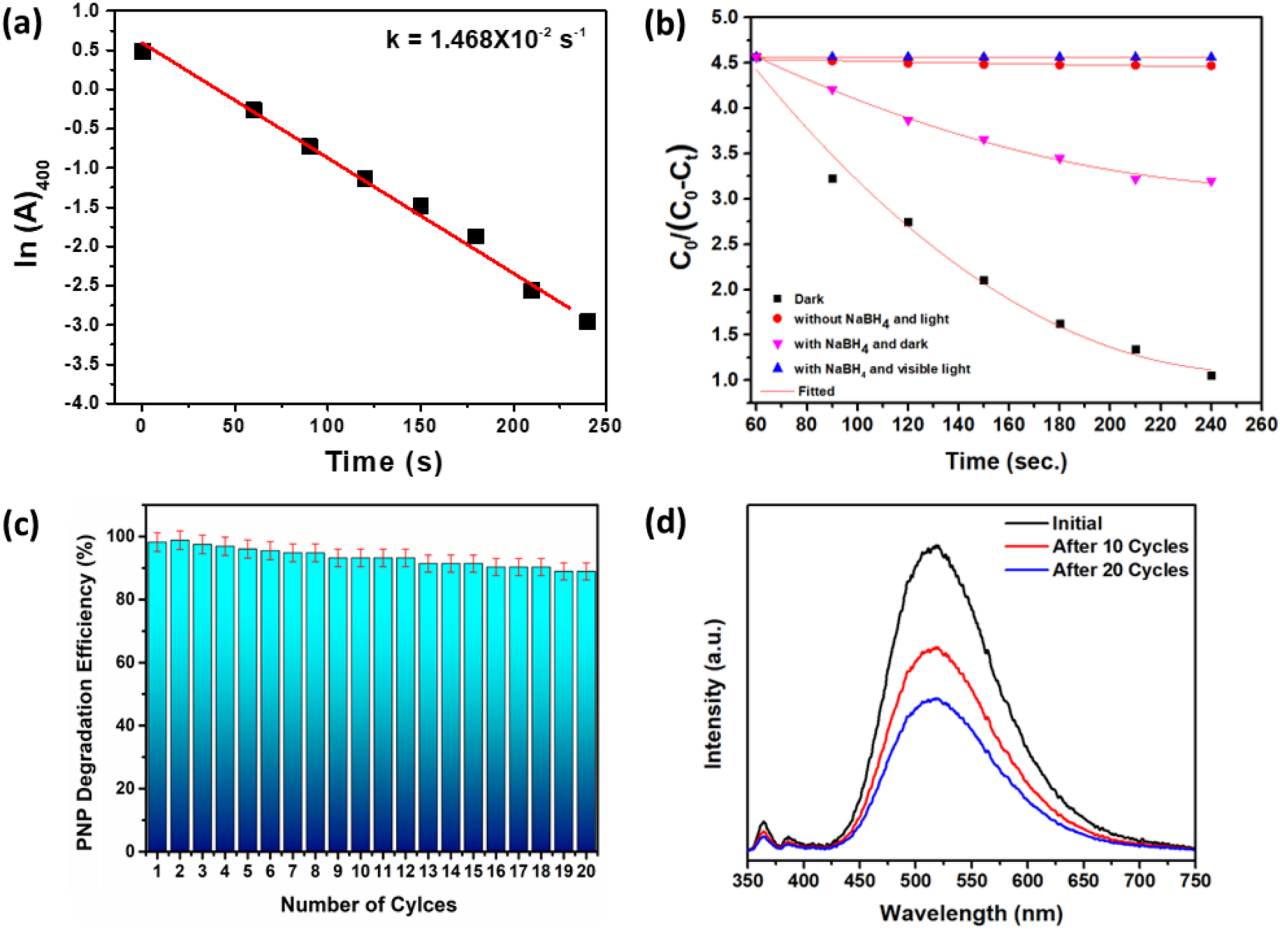
(**a**) ln(A) vs time plot for the rGO@ZnO (5 wt%) composite, (**b**) photocatalytic degradation plot under various conditions reflects the importance of NaBH_4_, (**c**) error bar cycle plot of the PNP reduction performance, (**d**) Room temperature photoluminescence spectra of rGO_5_@ZnO composite before and after catalytic use.

Figure 8a represents the linear correlation between ln (A) vs time reduction, indicating that the reaction is pseudo-first-order. The rate constant (k) calculated from the slope is k = 1.468 × 10^–2^ s^-1^. The effect of NaBH_4_ addition was assessed under various conditions, as illustrated in Fig. 8b. Notably, NaBH_4_ was found to significantly promote the reduction process under visible light. However, the reduction process was much slower in the dark, and no reduction activity was observed in the absence of light, even in the presence of NaBH_4_. Furthermore, the adsorption curve of PNP solution decreased inevitably, attributed to PNP's adsorption effect on the photo-catalyst’s surface. To investigate the reusability of the catalyst, the catalyst was subjected to 20 cycles of degradation efficiency assessment, as shown in Fig. 8c. The results indicated that the rGO@ZnO catalyst could maintain an average of 90% of degradation efficiency, thus confirming the potential of the catalyst for reuse. Furthermore, the composite's room temperature photoluminescence (PL) spectrum revealed several intra-band emissions, as shown in Fig. 8d. Notably, the PL spectrum was dominated by a strong visible emission in the green region, with a maximum emission band of approximately 514 nm, exclusively originating from the singly ionized oxygen vacancy defects of ZnO. Interestingly, the intense green emission band was progressively reduced during the composite's catalytic exposure under light, exhibiting almost a 50% decrement after the 20th cycle of composite usage. This finding indicates that the photocatalytic behavior of the composite is governed by the oxygen defects in the ZnO crystal, as supported by the XPS results (Fig. 5c).

### Plausible photocatalytic reduction mechanism of PNP using rGO@ZnO composite as a catalyst

In general, the process of photocatalytic degradation involves the excitation of nanoparticles by photons that possess energy equal to or greater than their bandgap. This results in charge separation and migration, leading to surface oxidation and reduction reactions^47^. When exposed to visible light, rGO can act as a photosensitizer and inject electrons into the conduction band of ZnO, causing the excited electron–hole pair to remain separated for a longer period of time. These electrons react with dissolved oxygen species (ROS) to produce superoxide radicals, as shown in Eqs. 3–7. Typically, a narrower band gap of the photocatalyst results in more efficient ROS generation and higher photocatalytic activity. However, an overly narrow band gap may lead to the formation of electron–hole recombination centers, reducing the photocatalytic efficiency. Therefore, in photocatalytic applications, researchers strive to optimize the band gap of the photocatalyst to achieve a balance between efficient ROS generation and reduced electron–hole recombination. The ROS generated, including e-, ·OH, ·O_2_^-^, etc., can effectively decompose PNP molecules when reacting with rGO_5_@ZnO. The composite's ability to absorb more visible light enables the creation of a higher concentration of free electrons that can participate in redox reactions and produce reactive oxygen species.
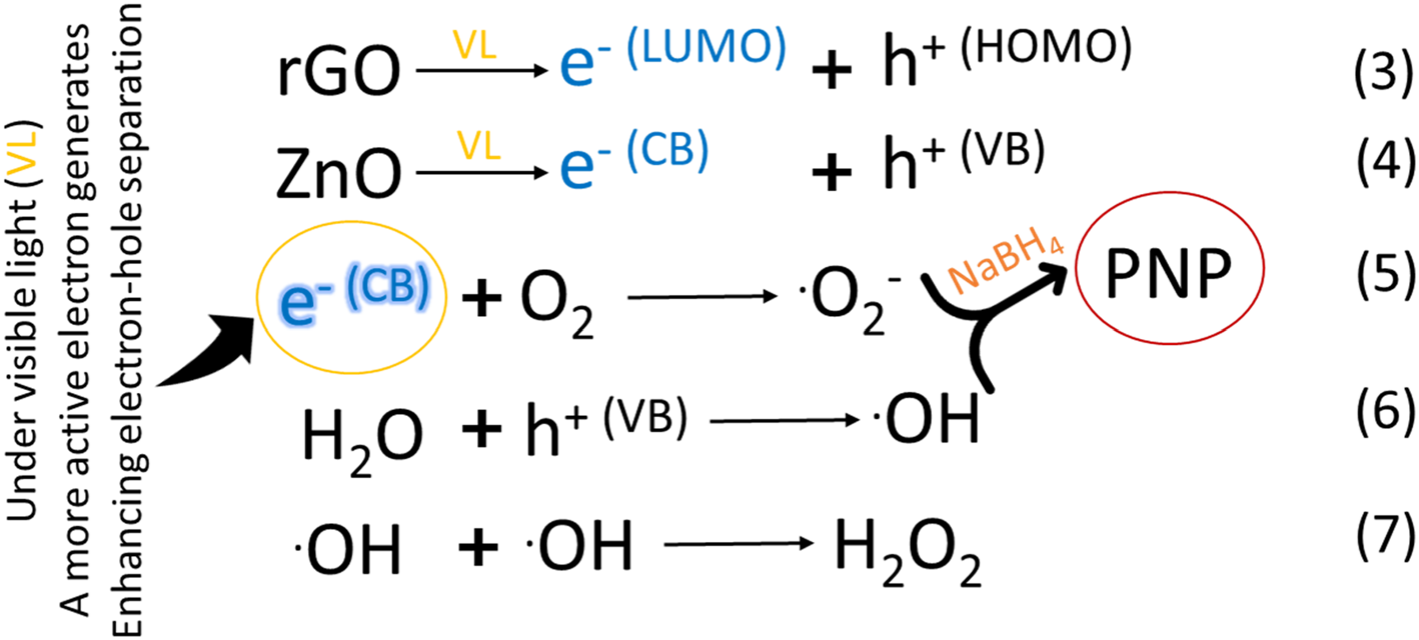


**Figure 9:**
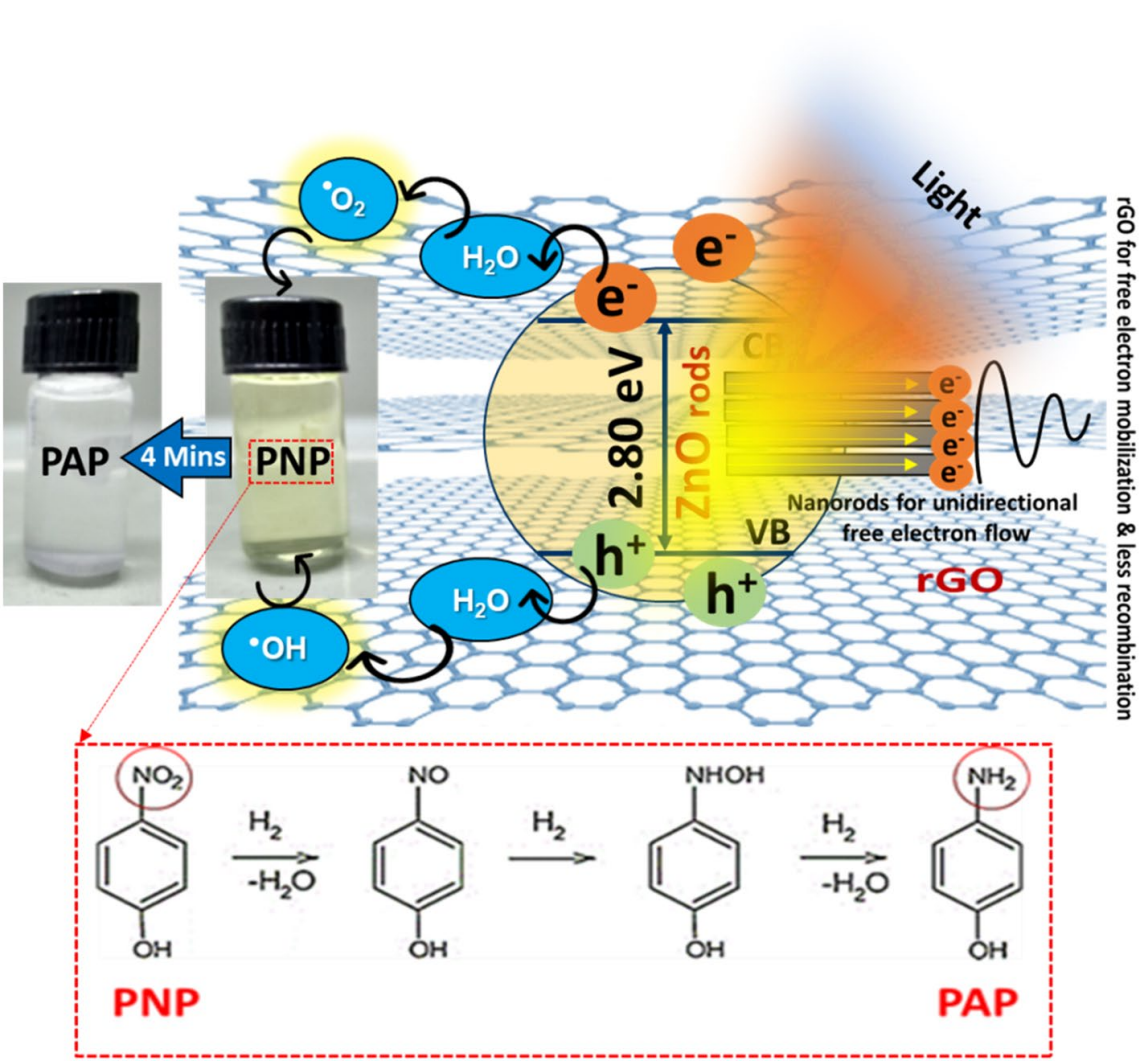
The schematic illustration of the photocatalytic reduction process of PNP to PAP utilizing rGO_5_@ZnO composite under visible light.

Reactive oxygen species (ROS) are formed when superoxide radicals react with water, as shown in Fig. 9. The figure depicts the valence band (VB) and conduction band positions of ZnO, along with the Highest Occupied Molecular Orbital (HOMO) and Lowest Unoccupied Molecular Orbital (LUMO) of rGO. The rGO@ZnO composite demonstrates visible light absorption, which leads to the excitation of electrons from the HOMO to the LUMO of rGO. Subsequently, the excited electrons from the LUMO of rGO move to the conduction band of ZnO across the interface, owing to energy matching and chemical synergy. The generated electrons produce ROS, thereby improving the photocatalytic efficiency. Moreover, electrons from the VB of ZnO can travel to the HOMO of rGO, leading to the efficient separation of the electron–hole pair (as shown in Fig. 9). This charge separation is essential in facilitating the rapid reduction of PNP. Additionally, Table 1 provides a brief comparison of the photocatalytic degradation capacity of various metal and metal oxide composite photocatalysts for 4-nitrophenol in industrial wastewater.

**Table 1:** Photocatalytic degradation performance of different metals/metal oxide containing composites for PNP compared with this work.

S. No	Photocatalyst material	The concentration of PNP, catalyst amount or concentration	Degradation efficiency	References
1	ZnO–rGO(6% Graphene)	NA	97.6% in 30 min	^30^
2	MoS_2_/ZnO	0.5 mM, 0.1 g	99% in 15 min	^48^
3	ZnO–Pt–RGO(Hydrothermal)	0.1 mM, 0.2 g	99% in 7 min	^49^
4	Ag/CuO	0.7 mM	More than 95% in 30 min	^50^
5	Cu_2_O/ZnO/rGO-10	0.3 g	98% in 90 min	^51^
6	NiWO_4_–ZnO–NRGO	0.1 mM, 0.1 mg	Nearly 98 in 80 s	^52^
7	Cu SMPs (Submicron particles)	0.14 mM, 3 mg	29 min	^53^
8	Ag–TiO_2_-SiO_2_–Fe_3_O_4_	2 × 10^-4^ M, 0.02 g	98% in 5 min	^54^
9	Se@ZnO/Gr-SZG	20 mg/L, 15 mg	99.22%	^55^
10	ZnO-Pt-RGO	0.1 mM, 2 mg	92% in 7 min	^49^
11	Maghemite/ZnO	0.2 mM, 0.2 mg	98.6% in 6–8 min	^56^
12	Cu_2_O/Cu-MOF/rGO	0.1 mM, 1 mg	90% in 7 min	^57^
13	rGO@ZnO	0.1 mM, 0.15 mg	98% in 4 min	**This work**

Significant values are in bold.

**Figure 10:**
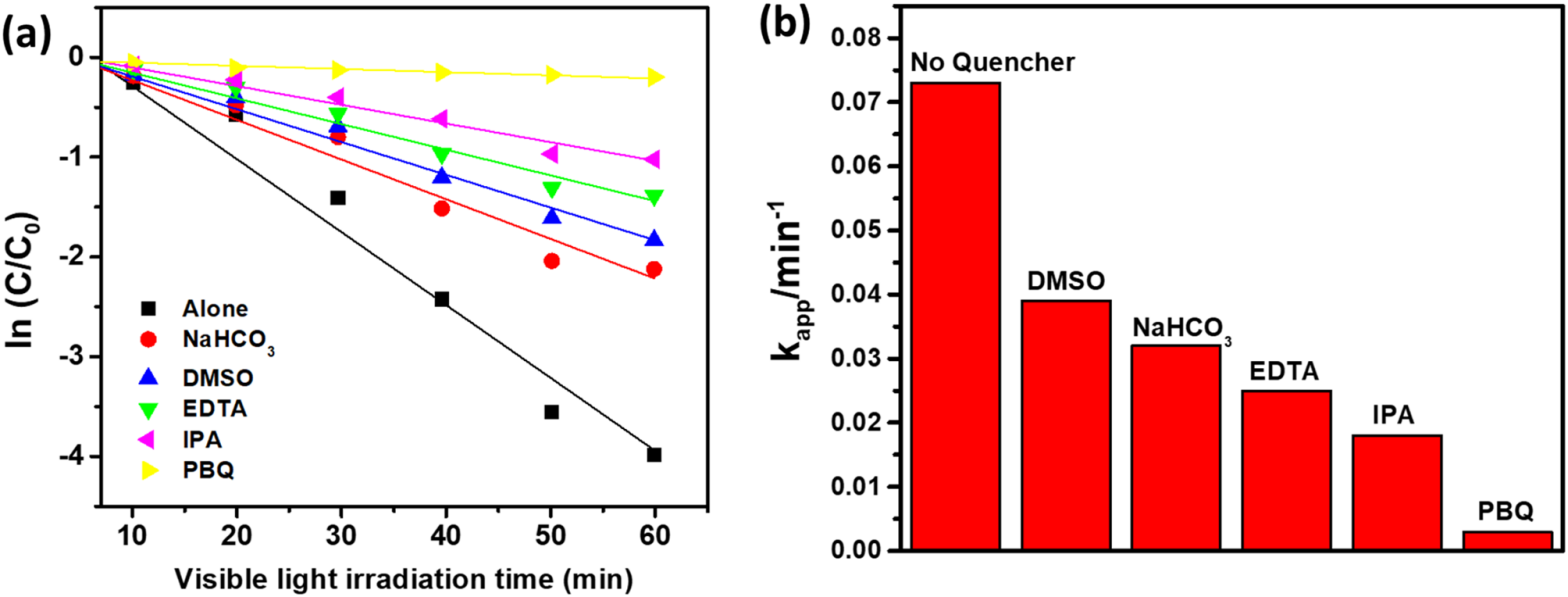
(**a**) Photocatalytic degradation kinetic plot for PNP in the presence of various scavengers as a function of visible light irradiation time, (**b**) different scavenger’s k_app_ values observed from the Langmuir–Hinshelwood equation.

In order to investigate the major reactive species such as e^-^ (electron), h^+^ (hole), O_2_^-^· (superoxide) and OH· (Hydroxyl) having relative importance during photocatalytic degradation under light illumination of the composite in the presence of various scavengers was performed^58^. Electrons and holes produced by photo-catalysis have efficient reduction and oxidation abilities during photocatalytic reactions. Establishing an actual degradation mechanism is prior to determining the contribution of actual radical or intermediate species towards the overall degradation process through a quenching experiment. During the experiment, NaHCO_3_ (as absorbed OH· quencher), dimethyl sulfoxide, DMSO (as e^-^ quencher), para benzoquinone, PBQ (as O_2_^-^ quencher), ethylene diamine tetra acetic acid, EDTA (as hole trapping agent) and isopropanol, IPA (as OH· quencher) were used as different scavengers for photocatalytic degradation of PNP^59–61^. Figure 10a indicates PNP’s degradation rate in the presence of different scavengers for the composite. It indicates a significant suppression in the presence of PBQ followed by IPA, whereas no significant result is observed for other scavengers. Again, from Fig. 10a it is observable that PBQ becomes the dominant species towards effective degradation of PNP into harmless by-products^62^.

The overall photo-degradation kinetics follows the first-order simplification of the Langmuir–Hinshelwood (L–H) mechanism, which is well established for photo-catalysis at low initial pollutant concentration (Ref.) The relevant equation is as follows (Eq. 8):

8 $$\mathrm{ln}\left(\frac{C}{{C}_{O}}\right)=-{k}_{app} t$$

where C/C_0_ is the ratio of the concentration of the PNP at initial and after various intervals of time, and k_app_ is the apparent first-order rate constant (time^-1^). Now the k_app_ value is determined from the gradient of the plot ln(C/C_0_) as *f* (t). If the free radical scavenged played a leading role in the photocatalytic degradation, the corresponding rate constant (k_app_) would be significantly reduced in the presence of an appropriate quencher^63,64^. Figure 10b signifies a bar diagram of k_app_ value resulting from different quenchers for phase pure and composite fibres, respectively. The corresponding L–H plot has been given inset of each bar diagram for both the photo-catalysts. For both cases, it is quite noticeable that PBQ contains extremely lower k_app_ followed by IPA, proving superoxide and hydroxyl radicals are the dominant reaction species during the photo catalytic reaction of PNP under visible light.

## Conclusions

The hydrothermal method was employed to synthesize rGO@ZnO composites by doping sonochemically synthesised ZnO nanorods on the surface of rGO. The homogeneous distribution of ZnO nanorods on rGO significantly enhanced the photocatalytic activity for the degradation of para-nitrophenol to amino-nitrophenol compared to pure ZnO and rGO catalysts. Among different weights of rGO (x = 0.5, 1, 3, 5, and 7 wt%), the composite rGO_x_@ZnO with 0.5 wt% of rGO achieved the highest degradation efficiency, indicating that the photocatalytic degradation of para-nitrophenol is influenced by the presence of an optimal weight percentage of rGO. A higher amount of rGO generates more oxygen imperfections during the reaction process, which can reduce the performance of photo degradation of the pollutants. The rGO_5_@ZnO nanocomposite has exhibited remarkable efficiency as a catalyst for the degradation of para-nitrophenol in industrial wastewater. Within a short period of 4 min under UV light, it achieved an impressive degradation rate of nearly 98%. This breakthrough highlights the potential of the nanocomposite for various applications in environmental conservation. Scavenger tests conducted revealed the impact of different reactive species on the photocatalytic activity. PBQ was found to suppress the degradation rate, indicating the dominance of superoxide radicals, while IPA suggested the significance of oxide radicals as another reactive species. Moreover, the reusability study demonstrated the composite's sustainability in the remediation of aromatic phenolic compounds. This finding opens doors to future modifications of its physical and chemical properties, which could enhance its effectiveness in degrading other harmful pollutants present in wastewater.

## Materials and methods

### Materials

Zinc acetate dihydrate (Zn(CH_3_CO_2_)_2_·2H_2_O), Ammonium hydroxide (NH_4_OH), graphite powder, potassium permanganate (KMnO_4_), sulfuric acid (98% H_2_SO_4_), hydrogen peroxide solution (H_2_O_2_), sodium borohydride (NaBH_4_), dilute hydrochloric acid (5% HCl), ethanol, para-nitrophenol (PNP) were obtained from Sigma Aldrich (now Merck Life Sciences, UK) and used without further purification. Deionized water (DI) was used throughout the experiments.

### Synthesis of ZnO nanorods

ZnO nanorods were synthesized by following the previous report. A homogeneous white precipitate of zinc hydroxide was formed during the addition of NH_4_OH for 2 h to reach the pH of 9.0 ± 0.5. Subsequently, the precipitate thus formed was centrifuged at ∼12,000 rpm and dispersed in distilled water to prepare a 0.3(w/v)% concentration of zinc hydroxide precursor solution followed by heating at 80 ± 5 °C for 6 h on a magnetic stirrer with constant stirring to induce the nucleation and growth of ZnO nanorods.

### Synthesis of reduced graphene oxide

Graphene Oxide (GO) was obtained from natural graphite powder via a modified Hummers method. Briefly, 0.25 g of graphite and 0.4 g of NaNO_3_ were taken in 12.5 mL of concentrated H_2_SO_4_ and stirred in an ice bath for 15 min. 2.0 g of KMnO_4_ was added slowly to obtain a greenish-purple suspension, followed by stirring for 6 h at 35 °C. The dark brown colour paste was diluted up to 100 mL with the slow addition of DI and sonicated for 20 min. 2 mL of 30% H_2_O_2_ was added dropwise to the solution. The golden-brown solution obtained was subjected to centrifugation and multiple washes with lukewarm DI water to achieve a pH of approximately 6. Subsequently, the product was left to air-dry for a period of 24 h. The resulting product was then dispersed in water through sonication for 30 min, followed by centrifugation at 3000 rpm for 15 min. The resulting supernatant was collected as graphene oxide (GO). To obtain reduced graphene oxide (rGO), the GO was further treated with aqueous NaBH_4_.

### Synthesis of rGO@ZnO composite

**Figure 11:**
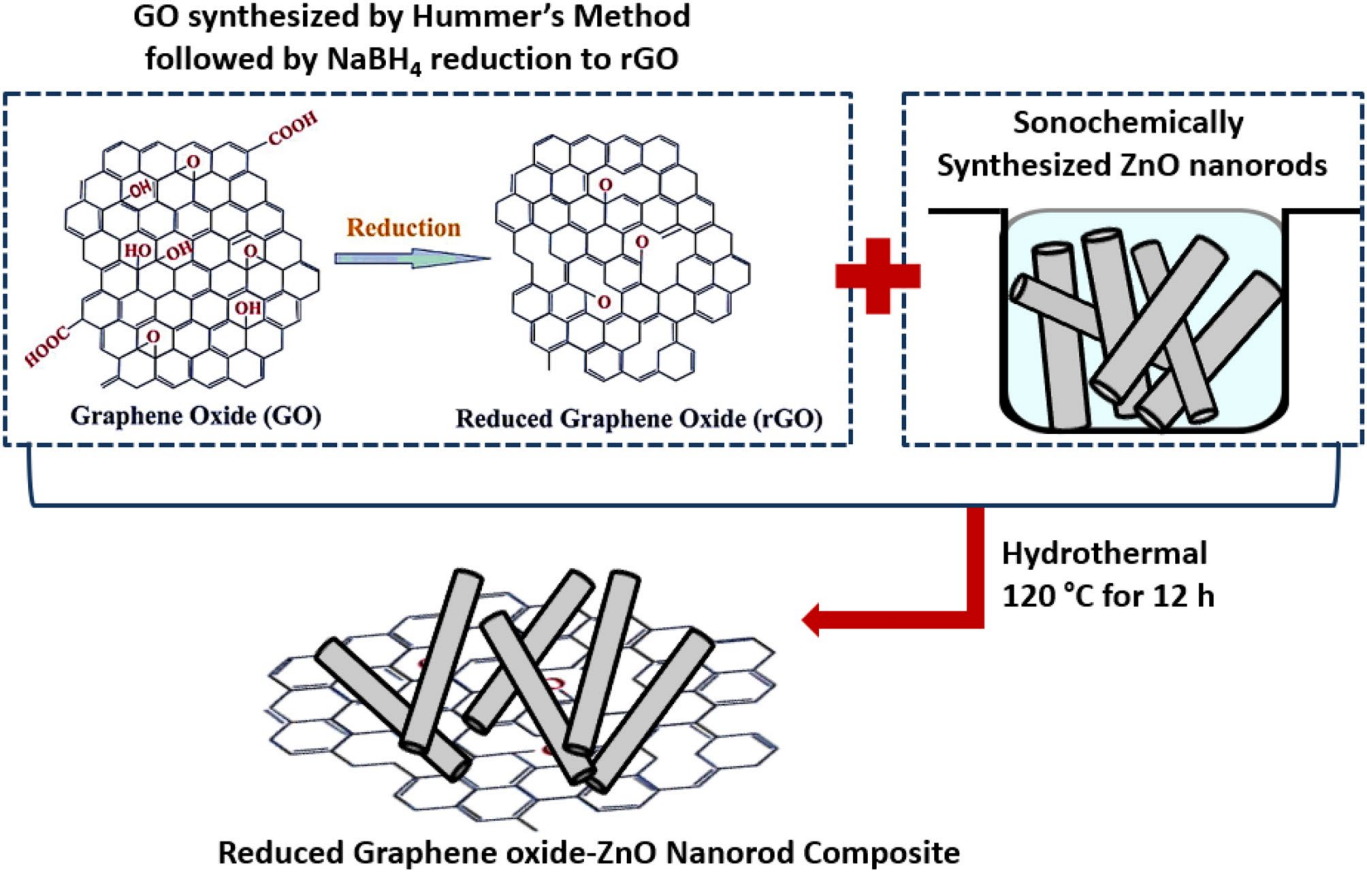
Schematic representation of the rGO@ZnO composite synthesis.

A homogeneous suspension was prepared by mixing 200 mg of rGO powder and 400 mg of ZnO nanorods in a mixture of 20 mL ethylene glycol and 20 mL DI water for 3 h. The resulting suspension was transferred to a 50 mL Teflon-lined stainless steel autoclave and maintained at a temperature of 120 °C for 12 h. Subsequently, the reaction system was allowed to cool down naturally to room temperature, followed by centrifugation at 7000 rpm for 10 min. The resulting black precipitate was washed multiple times with double distilled water, dried under vacuum for 12 h, and collected as a powder for further characterization. The synthesis process is depicted schematically in Fig. 11.

### Material characterizations

The X-ray diffraction (XRD) measurements were carried out on a Bruker D8 ADVANCE X-ray diffractometer (Cu Kα irradiation, 40 kV/40 mA). Fourier transform infrared (FTIR) adsorption spectra of the samples were recorded on a Nicolet 380 FTIR spectrometer using KBr. X-ray Photoelectron Spectroscopy (XPS) was carried out in a PREVAC EA15 system equipped with a 180° electrostatic hemispherical analyser (HSA), a 7 multi-channel detector and two multichannel plates, using a monochromatic Al K_α _radiation (1486.6 eV) operated at 12 kV and 25 mA X-ray source. The survey spectra were taken between 0 and 1200 eV with both survey and high-resolution scans recorded at a pass energy of 200 eV. Electron charge neutralisation was achieved using a PREVAC flood source FS40-PS with an ion gun current of 3 µA and an ion gun voltage of 0.2 V. All sample data was recorded at a pressure below 10^–9^ mPa. Raman spectra were acquired using a Renishaw In Via Reflex micro-Raman spectrometer with excitation of argon ion (514 nm) laser, and the spectra were collected with a resolution of 1 cm^-1^. A JEOL 2100 transmission electron microscope (TEM) at 200 kV. The pollutant removal efficiency was calculated from the UV–visible absorption spectroscopy measurements on a PerkinElmer LAMBDA 1050 UV/vis/NIR spectrophotometer. The photocatalytic experiments were performed at 27 ± 3 °C in an incubator shaker (Rivotek) with a shaking speed of 180 rpm and the ambient pH of the PNP solution (pH ∼ 7.1) without using any external acid/base. The photoluminescence spectroscopy measurement was conducted using the PL: FLS1000 Photoluminescence Spectrometer from Edinburgh Instruments.

### Photocatalytic experiment set-up and measurements

The photocatalytic properties of the synthesized nanomaterials were assessed utilizing a custom-designed photo reactor equipped with various light sources, including UV and visible light. The experimental procedure involved adding 0.15 mg of rGO@ZnO powder to a 4.8 mL aqueous solution of PNP (0.1 M) at ambient temperature. Subsequently, a freshly prepared solution of NaBH_4_ (0.25 mL, 0.1 M) was incrementally added to the mixture, followed by stirring the resulting solution in the dark until adsorption–desorption equilibrium was achieved. The progress of the reaction was periodically monitored using UV–Visible absorbance, and 3 mL of the solution was sampled in a quartz cuvette to measure the UV–Vis absorption spectra. Significantly, the absorption curve of the PNP solution exhibited a decline due to its adsorption onto the surface of the photo-catalyst. Additionally, the reusability of the catalyst was investigated for up to 20 cycles, demonstrating an average degradation efficiency of 90% and affirming the potential of rGO@ZnO for repeated utilization.

**Figure 12:**
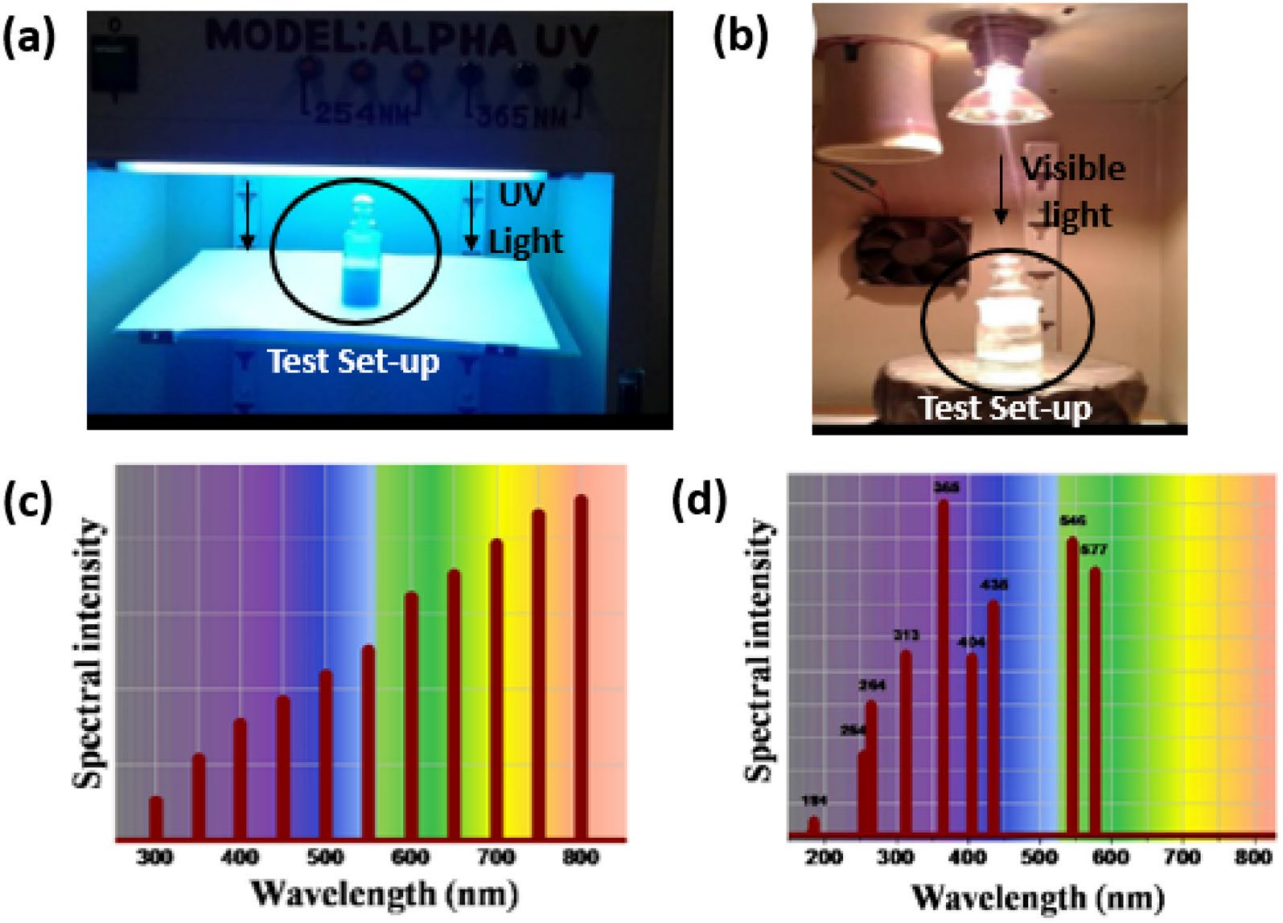
Photographs of the indigenously built photo reactors using (**a**) UV light and (**b**) visible light, respectively, schematic representation for the spectral region of (**c**) tungsten-halogen irradiation source and (**d**) high-pressure mercury lamp (250 W).

After the equilibration process, the photocatalytic mixture was subjected to different light sources. Monochromatic UV lamps of UVLS-28 EL Series and Phillips (8W) emitting λ_365_ nm and λ_254_ nm were used to conduct experiments with UV light (Fig. 12a). A 250W halogen-tungsten lamp with an air-cooling system served as a visible light source (Fig. 12b). The spectral distribution of the light sources is illustrated in Fig. 12c and Fig. 12d. To eliminate the UV region, an optical cut filter with a wavelength of λ ≥ 420 nm was attached to it. For simulating solar irradiation, a 250W high-pressure mercury lamp equipped with an air-cooling system was used as the irradiation source without any optical cut-off filter. The photocatalytic mixture was collected at regular irradiation intervals, centrifuged, and UV–visible absorbance spectra were monitored using the Shimadzu UV-3600 spectrophotometer. The photocatalytic activity of the efficient sample was tested at least three times to confirm the photo-stability of the sample. Controlled experiments were conducted under identical conditions for comparative purposes. The extent of photocatalytic degradation was evaluated using Eq. 4.

9 $$Degradation \left(\%\right)=\frac{{C}_{O}-{C}_{t}}{{C}_{o}}100$$

C_o_ and C_t_ represent the initial absorbance and absorbance after "t" min reaction time at a λ_max_ of the target pollutant solution.

## Supplementary Information

Supplementary Information.

## Data Availability

All data generated or analysed during this study are included in the article that is available from the corresponding author.
