# Supplementary material for: Effectual visible light photocatalytic reduction of para-nitro phenol using reduced graphene oxide and ZnO composite
Source: Sci Rep. 2023 Jun 12;13:9521. doi: 10.1038/s41598-023-36574-7 (PMC10261142; doi:10.1038/s41598-023-36574-7)
Supplement: Supplementary file 1 — Supplementary Information. [file 41598_2023_36574_MOESM1_ESM.docx]

**Supporting Document**

**Effectual Visible Light Photocatalytic Reduction of Para-Nitro Phenol using reduced graphene oxide and ZnO composite**

Sasireka Velusamy,^a#^ Anurag Roy,^a#*^ Ezrah Mariam,^b^ Satheesh Krishnamurthy,^b^ Senthilarasu Sundaram,^c*^, Tapas K. Mallick^a^

^a^ Solar Energy Research Group, Environment and Sustainability Institute (ESI), Faculty of Environment, Science and Economy, Penryn Campus, University of Exeter, Cornwall, TR10 9FE, United Kingdom.

^c^ School of Engineering and Innovation , The Open University, Milton Keynes, MK7 6AA, United Kingdom.

^c^ Cybersecurity and Systems Engineering, School of Computing, Engineering and the Built Environment, Edinburgh Napier University, Edinburgh, EH10 5DT, United Kingdom.

* Corresponding authors

E-mail: [s.sundaram@napier.ac.uk](mailto:s.sundaram@napier.ac.uk) and [A.Roy30@exeter.ac.uk](mailto:A.Roy30@exeter.ac.uk)

^#^ These authors contributed equally.


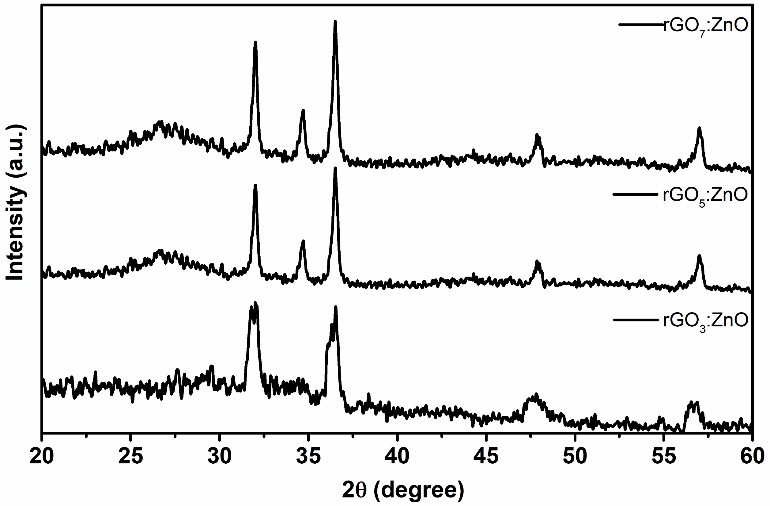


Figure. S1. XRD patterns of the synthesized rGOx@ZnO (x= 3, 5, and 7 wt %) composite samples.


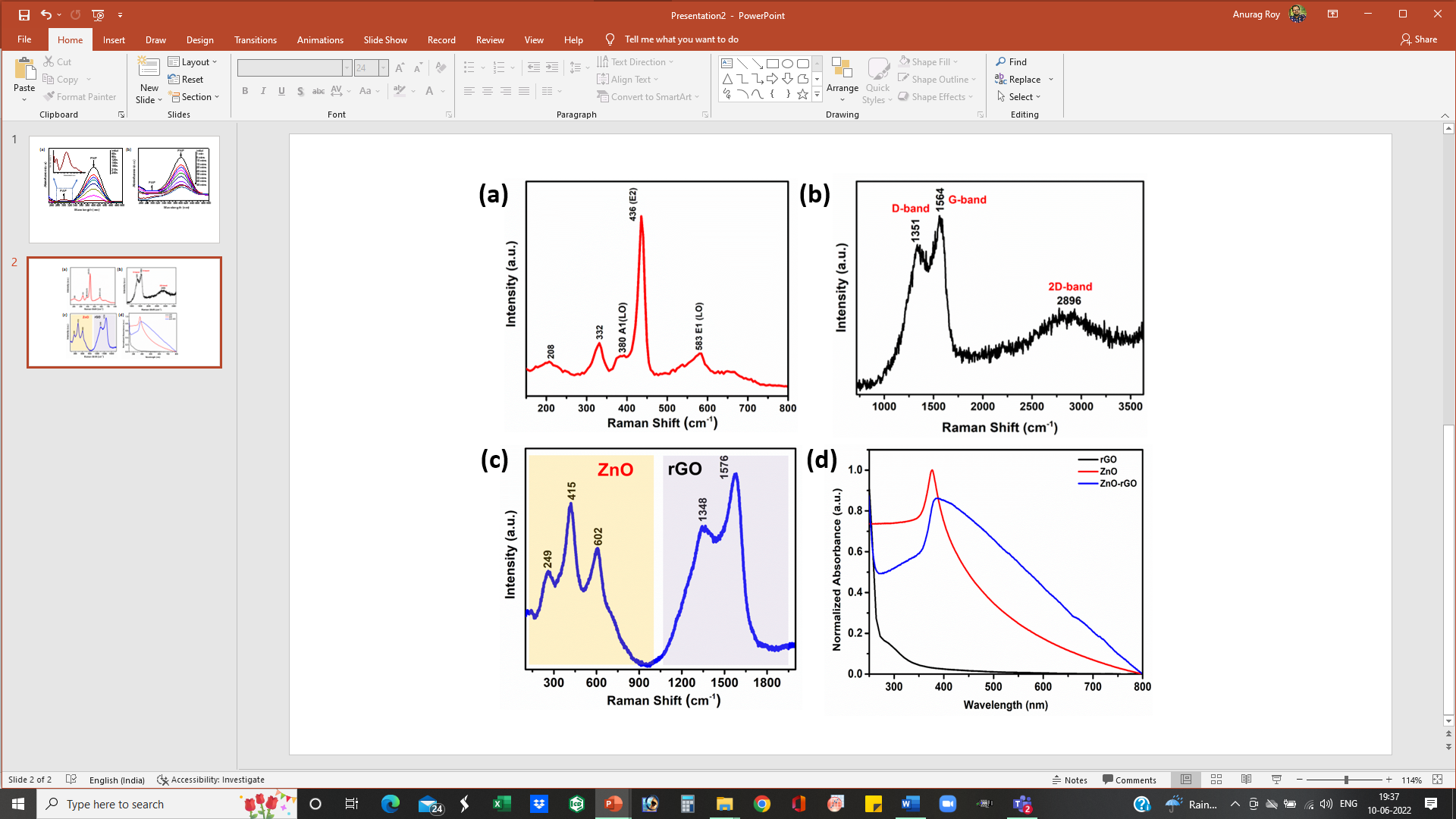

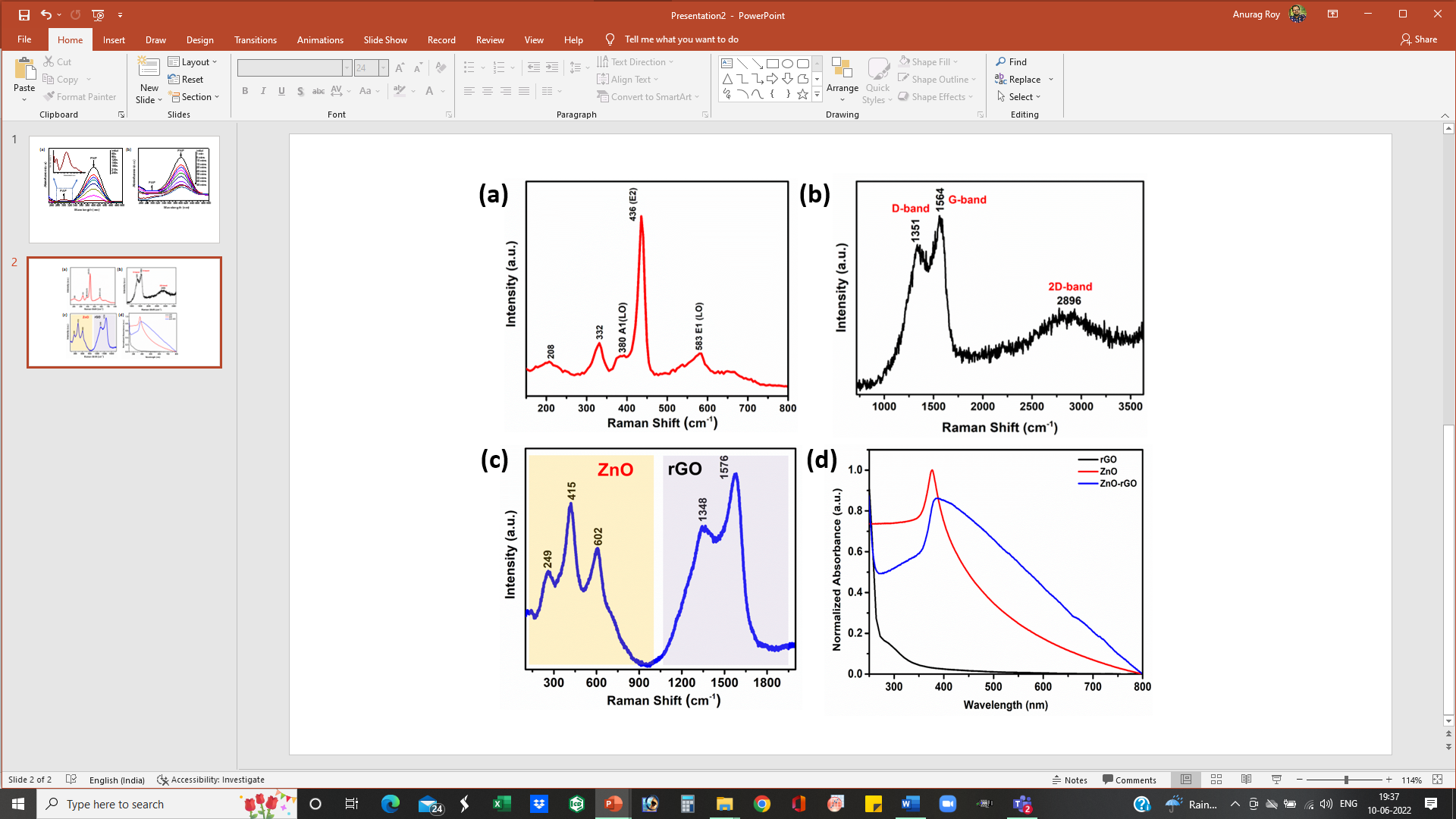


**Figure. S2**. Raman spectrum of synthesized (**a**) ZnO, (**b**) rGO, respectively.


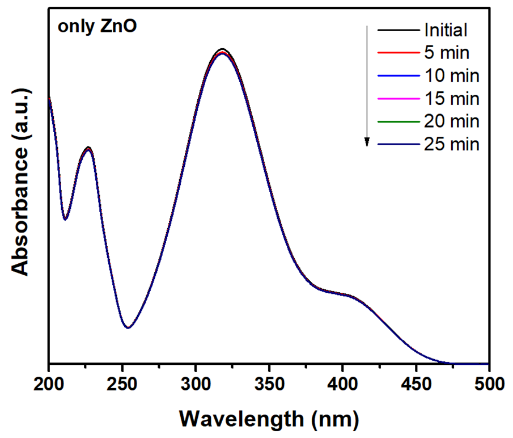


**Figure. S3** Photocatalytic reduction characteristics of PNP under UV light or visible light in the presence of ZnO alone.
